# C-Phycocyanin Prevents Oxidative Stress, Inflammation, and Lung Remodeling in an Ovalbumin-Induced Rat Asthma Model

**DOI:** 10.3390/ijms25137031

**Published:** 2024-06-27

**Authors:** Zayra Mundo-Franco, Julieta Luna-Herrera, Jorge Ismael Castañeda-Sánchez, José Iván Serrano-Contreras, Plácido Rojas-Franco, Vanessa Blas-Valdivia, Margarita Franco-Colín, Edgar Cano-Europa

**Affiliations:** 1 Laboratorio de Metabolismo I, Departamento de Fisiología, Escuela Nacional de Ciencias Biológicas, Instituto Politécnico Nacional, Mexico City 07738, Mexico; zmundof1500@alumno.ipn.mx (Z.M.-F.); projasf@ipn.mx (P.R.-F.); mfrancoc@ipn.mx (M.F.-C.); 2 Laboratorio de Inmunoquímica II, Departamento de Inmunología, Escuela Nacional de Ciencias Biológicas, Instituto Politécnico Nacional, Mexico City 11340, Mexico; 3 Departamento de Sistemas Biológicos, Universidad Autónoma Metropolitana, Unidad Xochimilco, Mexico City 04960, Mexico; jcastanedas@correo.xoc.uam.mx; 4 Section of Nutrition, Division of Digestive Diseases, Department of Metabolism, Digestion and Reproduction, Faculty of Medicine, Hammersmith Campus, Imperial College London, Du Cane Road, London W12 0NN, UK; j.serrano-contreras@imperial.ac.uk; 5 Laboratorio de Neurobiología, Departamento de Fisiología, Escuela Nacional de Ciencias Biológicas, Instituto Politécnico Nacional, Mexico City 07738, Mexico; vblasv@ipn.mx

**Keywords:** asthma, C-phycocyanin, ovalbumin, airway remodeling, inflammation

## Abstract

Asthma is a chronic immunological disease related to oxidative stress and chronic inflammation; both processes promote airway remodeling with collagen deposition and matrix thickening, causing pulmonary damage and lost function. This study investigates the immunomodulation of C-phycocyanin (CPC), a natural blue pigment purified from cyanobacteria, as a potential alternative treatment to prevent the remodeling process against asthma. We conducted experiments using ovalbumin (OVA) to induce asthma in Sprague Dawley rats. Animals were divided into five groups: (1) sham + vehicle, (2) sham + CPC, (3) asthma + vehicle, (4) asthma + CPC, and (5) asthma + methylprednisolone (MP). Our findings reveal that asthma promotes hypoxemia, leukocytosis, and pulmonary myeloperoxidase (MPO) activity by increasing lipid peroxidation, reactive oxygen and nitrogen species, inflammation associated with Th2 response, and airway remodeling in the lungs. CPC and MP treatment partially prevented these physiological processes with similar action on the biomarkers evaluated. In conclusion, CPC treatment enhanced the antioxidant defense system, thereby preventing oxidative stress and reducing airway inflammation by regulating pro-inflammatory and anti-inflammatory cytokines, consequently avoiding asthma-induced airway remodeling.

## 1. Introduction

Bronchial asthma is a heterogeneous allergic disease characterized by chronic airway inflammation diagnosed by a history of wheezing, breathlessness, chest tightness, and coughing varying over time, intensity, and expiratory airflow limitation [1]. Two endotypes, or pathophysiological mechanisms, have been reported: T2-low and T2-high asthma. The first is associated with airway neutrophilia, corticosteroid resistance, Th1/Th17 polarization, and the absence of type 2 immune production. Moreover, Th1 cells, Th17 cells, and M1 macrophages involved in this endotype produce the IL-1β, IL-6, IL-17, IFN-γ, and TNF-α responsible for the recruitment and activation of inflammatory cells during asthma attacks [2]. On the other hand, T2-high is the classical asthma presentation with large amounts of cytokines, such as IL-4 (produced by mast cells), IL-5 (produced by eosinophils), and IL-13, facilitating the synthesis of allergen-specific IgE and the accumulation of inflammatory cells, such as eosinophils, mast cells, basophils, and M2 macrophages. The degranulation of mast cells and basophils releases various cytokines, chemokines, vasoactive amines, and lipid mediators, promoting inflammation, bronchoconstriction, vascular permeability, and exacerbating mucus production [3]. In asthma, continuing interaction with allergens induces chronic inflammation and airway remodeling, which is not fully reversible, causing an accelerated and progressive loss of lung function over time. Recognition of disease-associated molecular patterns DAMPS by immune-competent cells, such as tissue endothelial cells and macrophages, is favored during asthma exacerbation triggers the inflammatory response primarily by and adaptive immune reactions toll-like receptors (TLRs), especially lung endothelial TLR4 recognizes several exogenous ligands and activates a series of inflammatory cascades NFκB-dependent like macrophage activation and is involved in outward remodeling, promoting the inflammatory process [4,5].

Remodeling in asthma induces structural change and dysfunction of airway smooth muscle, respiratory epithelium, endothelium, and mucus glands [6,7]. The treatment of this disease involves beta-agonists, muscarinic antagonists, and inhaled corticosteroids [8]. Although these drugs effectively manage acute asthma symptoms, only corticosteroids, such as methylprednisolone (MP), partially prevent the airway remodeling process [9]. MP diffuses passively across the cellular membrane and binds to the intracellular glucocorticoid receptor, resulting in the repression of proinflammatory genes inhibited by blocking the function of NFκB. MP also suppresses the synthesis of cyclooxygenase 2 (COX2), reducing the production of prostaglandins [10,11].

Asthma affects 300 million people worldwide, causing a substantial and growing burden of morbidity and mortality, especially in low- and middle-income countries. The prevalence of asthma is estimated to be approximately 11.5% based on population studies and is highest in the African region (13.2%) and lowest in the Americas (10.0%). The Global Initiative for Asthma recommends that all patients always receive inhaled corticosteroids and long-acting beta-agonists.

Asthma accounts for significant direct and indirect healthcare costs, estimated over USD 80 billion per year [12]. Asthma-related costs impose a high burden on all countries and differ depending on factors such as gross domestic product (GDP), type of healthcare system, and public health resources. In Indonesia, Thailand, and Vietnam, the costs for asthma treatment involving hospitalization, healthcare visits, home visits, and medication per patient reach annual costs of USD 215,570; USD 41,670 and USD 373, respectively [13]. In Germany the costs for patients are between EUR 6618.90 and EUR 9712.38 in the baseline period and EURO 22,832.33 and EURO 13,717.74 in the follow-up period [14]. Unfortunately, this disease perpetuates poverty in Mexico because not all patients can afford the recommended therapy due to the cost (~USD 1000 out of pocket per year). Regrettably, this is often at the expense of food and other basic living requirements. However, short-acting beta-agonists are affordable to reduce asthmatic crises without avoiding airway remodeling, and the possibility of developing chronic obstructive pulmonary disease is elevated [15]. So, it is crucial to create inexpensive treatment strategies to reduce the asthma crisis and its complications. In this context, nutraceutical therapies could be an alternative.

Nutraceuticals provide therapeutic health benefits because they are a food or part of a food with pharmacological properties [16]. Nutraceuticals have several dosage forms, such as powder, tablet, capsule, or liquid. One of the most studied nutraceuticals is Spirulina (*Arthrospira maxima*), which has anti-inflammatory activity in animal models of asthma and human clinical trials due to it possessing nutraceutical molecules, such as vitamins, polyunsaturated fatty acid and pigments such chlorophyll a, β-carotene, zeaxanthin, and phycobiliproteins (C-phycocyanin (CPC) and allophycocyanin) [17].

CPC is a 120 kDa protein with an important light-yielding pigment that is intermittently systematized in the cyanobacterial species [18]. It has numerous applications in the field of biotechnology and the drug industry; it possesses variable beneficial properties as a treatment, including antioxidant [19,20], anti-inflammatory [21], organic multi-protector [22,23] and anti-cancerogenic [24]. Therefore, it has been proposed that CPC acts as a prodrug activated in the gastrointestinal tract and metabolized into phycocyanobilin (PCB) [25]. In addition, PCB is the biological molecule responsible for other nutraceutical properties such as nephroprotective and anti-inflammatory [17,25]. However, CPC’s complete pharmacological action as an asthma treatment is still unknown. Thus, this research aimed to evaluate if the CPC in *Arthrospira maxima* reduces the inflammatory process, oxidative stress, and lung remodeling in ovalbumin-challenged asthmatic rats.

## 2. Results

### 2.1. Effect of CPC on Clinical Pathology Markers Related to Asthma

**Figure 1:**
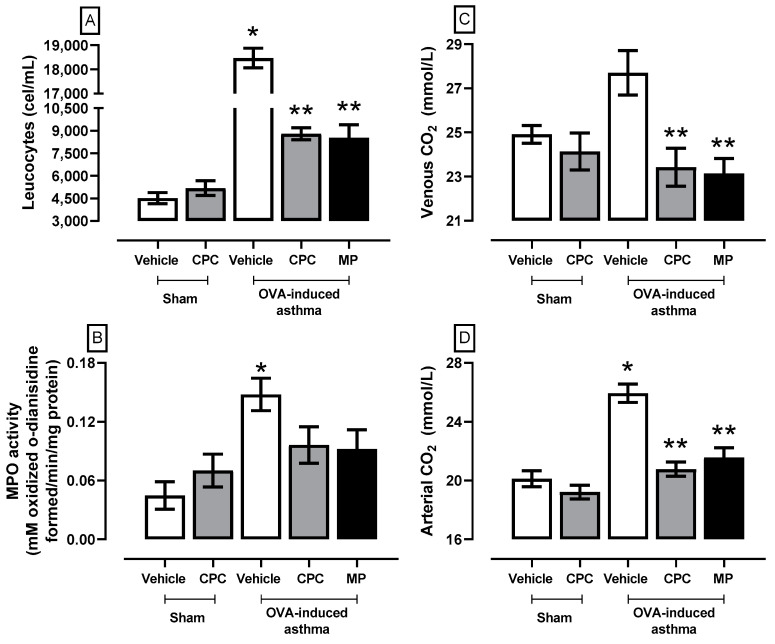
Effect of C-phycocyanin (CPC) on leucocytes (**A**), pulmonary myeloperoxidase activity (**B**), venous CO_2_ (**C**), and arterial CO_2_ (**D**) of ovalbumin (OVA)-induced asthma in rats. Values represent the mean ± SEM. * *p* < 0.05 compared to the sham-vehicle group at the same time and ** *p* < 0.05 compared to the OVA-induced asthma group. One-way ANOVA and Student–Newman–Keuls post hoc test.

To check the asthmatic status of the rats and validate the model, after immunization and administration of OVA and the consequent treatments with the drug of choice (MP) and the novel alternative treatment (CPC), Figure 1 shows clinical pathology markers of asthma that rise in the OVA-induced asthma group, where there is leukocytosis, an increase in venous and arterial CO_2_ concentration and increased pulmonary MPO activity. On the other hand, CPC and MP denoted a decrease in the markers that were indistinct between the drug and the nutraceutical. Specifically, both prevent the increase of leukocyte presence (~50%), MPO activity (~35%), arterial CO_2_ concentration (~15%), and venous CO_2_ concentration (~17%).

### 2.2. Effect of CPC on Asthma-Induced Oxidative Stress and Cellular Damage

**Figure 2:**
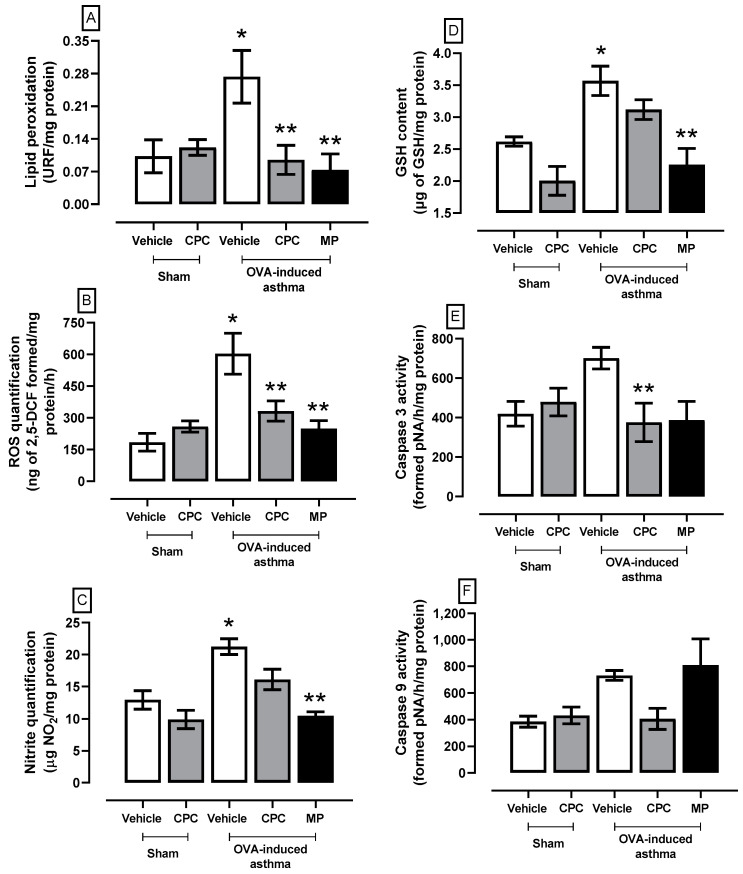
Effect of C-phycocyanin (CPC) on lipid peroxidation (**A**), ROS quantification (**B**), nitrite quantification (**C**), GSH content (**D**), and caspase 3 (**E**) and caspase 9 activities (**F**) in the lung of ovalbumin (OVA)-induced asthma in rats. Values represent the mean ± SEM. * *p* < 0.05 compared to the vehicle-sham group and ** *p* < 0.05 compared to the OVA-induced asthma group. One-way ANOVA and Student–Newman–Keuls post hoc test.

The lung REDOX environment and the apoptosis markers evaluation are illustrated in Figure 2. The asthmatic rats showed the highest levels of reactive oxygen species (ROS), lipid peroxidation (LP), nitrites (NOS), reduced glutathione (GSH), and caspase 3 and 9 activities. The CPC treatment partially prevented asthma-induced oxidative stress (ROS 65%, LP 45%) and prevented cellular damage by reducing the activities of caspases 3 and 9 by about 45% compared to the OVA group. However, the treatment with MP had a better response against oxidative stress and cellular damage markers as it prevents the enhancement of oxidative stress markers (LP ~73%, ROS ~58%, GSH ~37%, and nitrites ~50%), and caspase 3 (~45%).

### 2.3. Histopathological Study of the Lung of Ovalbumin-Challenged Asthmatic Rats

**Figure 3:**
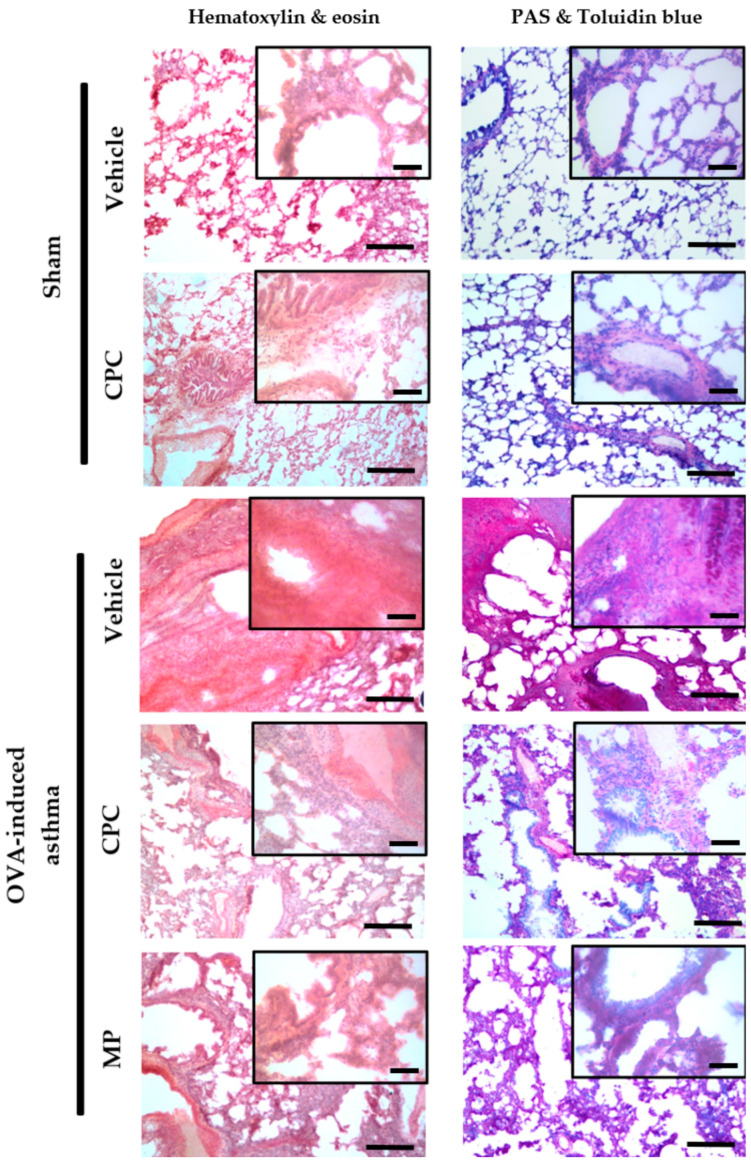
Effect of C-phycocyanin (CPC) on asthma-induced pulmonary remodeling process in the lung. Representative micrographs of lung tissues stained by hematoxylin–eosin (H&E) and periodic acid stain (PAS)-blue toluidine. The lower right bar of the big photomicrographs represents 250 μm and the little one represents 100 μm.

Figure 3 depicts the histopathological study of the lung. In sham groups, normal bronchiolar and alveoli cytoarchitecture was observed. It is characterized by alveolar ducts open to alveoli from the respiratory bronchiole, which is covered by low columnar ciliated cells. Blue toluidine and PAS stain shows the basal membrane without goblet cells. Also, most areas of the alveolar lumen are covered by flat cells, such as pneumocytes type I and II (stain H&E). Meanwhile, the asthmatic group without treatment promoted airway remodeling, which is an ongoing structural change that leads to thickened airway walls due to goblet cell hyperplasia (demonstrated by blue toluidine and PAS stain), subepithelial collagen deposition with increased thickness of the reticular basement membrane, and smooth muscle, as well as infiltration of multiple immunological cells promoting the mucus production. Additionally, the asthmatic rats treated with CPC and MP showed similar histopathological signs, such as mild airway remodeling process and lower inflammation than the asthmatic rats. Although both treatments prevented severe inflammation, only the MP treatment reduced the mucus production compared with the CPC treatment.

### 2.4. Participation of the TLR4/NFκB/COX2/NOS2 Pathway and Macrophages on OVA-Induced Asthma

**Figure 4:**
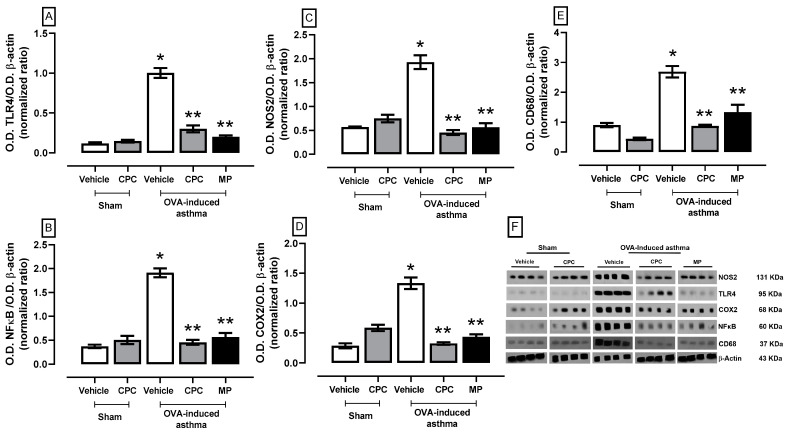
Effect of C-phycocyanin (CPC) on inflammation markers (**A**) TLR4, (**B**) NFκB, (**C**) NOS2, (**D**) COX2, and (**E**) CD68 in the lung of ovalbumin (OVA)-induced asthma in rats. (**F**) expression blot of all proteins by Western blotting technique. Values represent the mean ± SEM. * *p* < 0.05 compared to the vehicle-sham group and ** *p* < 0.05 compared to the OVA-induced asthma group. One-way ANOVA and Student–Newman–Keuls post hoc test.

Upon recognition of the DAMPs, TLRs trigger the production of pro-inflammatory mediators, helping to eradicate infection; in asthma, the OVA particles in constant contact with the lung cells trigger a chronic inflammatory response. Figure 4 illustrates the inflammation process of the lungs in asthma; the results showed an overexpression of approximately twice to fourfold in the most important signaling proteins of the TLR4/NFκB/COX2/NOS2 pathway that concludes in the overexpression and production of proinflammatory cytokines and CD68, the membrane protein exclusive of macrophages. Meanwhile, treatment with CPC and MP avoids the overexpression of all evaluated proteins, mitigating the inflammation process.

### 2.5. Effect of CPC on the Expression of Collagen1a1 and αSMA as Lung Remodeling Markers

**Figure 5:**
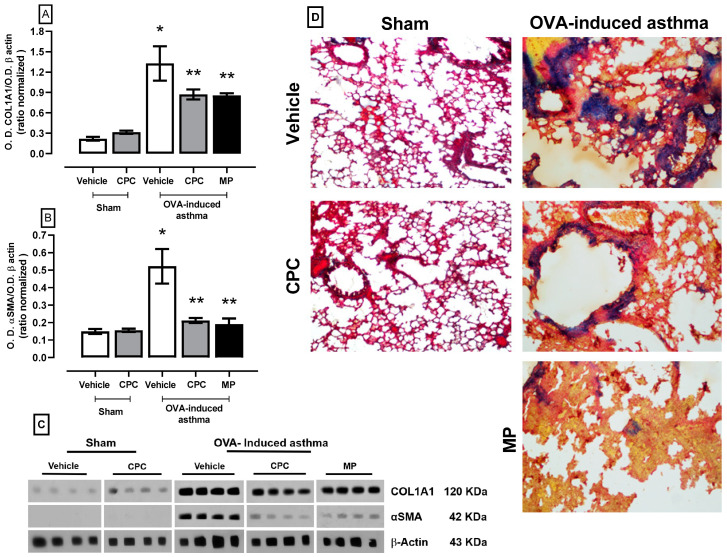
Effect of C-phycocyanin (CPC) on (**A**) αSMA, (**B**) COL1A1, and fibrosis in the lung of ovalbumin (OVA)-induced asthma in rats. (**C**) expression blot of all proteins by Western blotting technique, (**D**) representative microphotography of lung tissues stained by Masson’s trichrome stain, the lower right bar of the big photomicrographs represents 250 μm. Values represent the mean ± SEM. * *p* < 0.05 compared to the sham-vehicle group and ** *p* < 0.05 compared to the OVA-induced asthma group. One-way ANOVA and Student–Newman–Keuls post hoc test.

Figure 5 illustrates the markers expression of the fibrosis process due to asthma, showing an over-expression of the collagen1a1 and αSMA corroborated fibrosis and remodeling with clusters of collagen fibers in the bronchial epithelium by Masson’s trichrome stain where collagen is stained blue, and smooth muscle tissue is stained red. The treatment with CPC and MP denotes the down expression of collagen1a1 and αSMA at approximately 50%, avoiding the remodeling process and preventing pulmonary fibrosis.

### 2.6. Effect of CPC on a Cytokine Expression Related to the Inflammatory Process in Asthma

**Figure 6:**
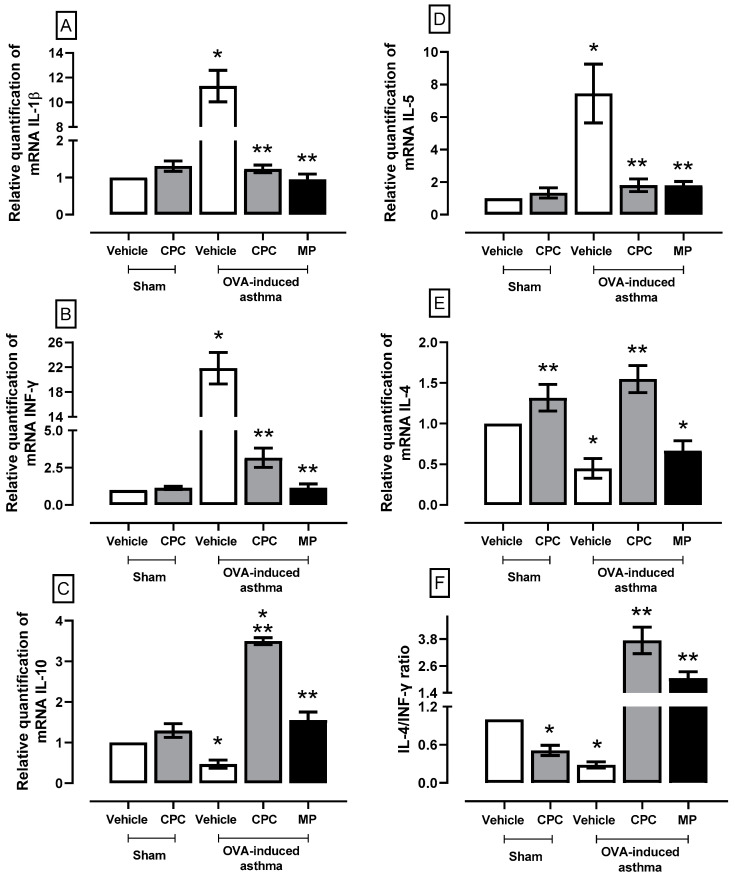
Effect of C-phycocyanin (CPC) on mRNA synthesis of cytokines such as (**A**) IL-1β, (**B**) INF-γ, (**C**) IL-10, (**D**) IL-5, (**E**) IL-4, and (**F**) IL-4/INF-γ ratios in the lung of ovalbumin (OVA)-induced asthma in rats. Values represent the mean ± SEM. * *p* < 0.05 compared to the sham-vehicle and ** *p* < 0.05 compared to the OVA-induced asthma group. One-way ANOVA and Student–Newman–Keuls post hoc test for all cytokines except for IL-4/INF-γ ratio were analyzed using Kruskal–Wallis one way-ANOVA and Student–Newman–Keuls post hoc test.

Figure 6 shows that OVA increases the synthesis of pro-inflammatory cytokines in the lung: IL-1β (~11%), INF-γ (~22%), and IL-5 (~7%). Nevertheless, CPC and MP treatments lower the synthesis of these pro-inflammatory cytokines and increase the synthesis of anti-inflammatory cytokines IL-10 (~8%) and IL-4 (~3%) compared to asthma-induced groups. Asthma generally enhances the IL-4/INF-γ ratio fourfold compared to the sham group.

## 3. Discussion

Asthma is a chronic immunological disease related to oxidative stress and chronic inflammation that significantly affects individuals’ quality of life. However, in middle- and lower-income countries, proper medication management is not accessible to all patients. Asthma accounts for significant direct and indirect health care costs, estimated over USD 80 billion annually worldwide [12]. In Mexico, asthma is considered a public health problem because it affects 3.35 million people, with 606.0 thousand new incident cases and 1655 deaths in 2019 [26]. Also, in a study of a Mexican third-level hospital (Instituto Nacional de Enfermedades Respiratorias, INER), specialized in pulmonary disease, the estimated cost for direct medical treatment per patient was about MXN $43,813.92 (USD $2613.12), which corresponds to 9.18% of its total budget in 2022 [27]. Therefore, nutraceuticals could become a co-treatment for asthma, a delayer for its long-term complications, reducing the impact on patient quality of life. One option is the treatment with CPC from *A. maxima*, which has an immunomodulatory action because, in the model of OVA-induced asthma, we found diminished pulmonary oxidative stress, inflammation, and airway remodeling like corticosteroid treatment.

The model of OVA-induced asthma reproduces the most common endotype (T2-high), promoting airway obstruction, hypoxia, and inflammation through Th2-response. This allows the airway remodeling process characterized by collagen deposition and matrix thickening at the expense of hyperplasia of smooth muscle and goblet cells. This pathology should not be underestimated, as it can result in irreversible changes in the airway structure, which leads to airflow obstruction and long-term loss of lung function [28]. Evidence suggests that chronic and uncontrolled inflammation in asthma arises not only from increased or repetitive exposure to allergens, leading to excessive airway inflammation, but also from the uncontrolled and inadequate activation of pro-resolving pathways [29]. Thus, we hypothesize that CPC is more than a common antioxidant that prevents oxidative stress and inflammation but a prodrug that is metabolized along the gastrointestinal tract to be absorbed as chromo-peptides and/or the linear tetrapyrrole, the phycocyanobilin (PCB), which has a biological effect on several immunological blanks resolving the inflammation induced by OVA challenge in rats.

The CPC’s antioxidant properties have been reported to rely on its activity as a nucleophilic compound that neutralizes reactive oxygen species and free radicals. In the case of asthma, CPC can neutralize the excessive production of free radicals during allergen-induced inflammation and avoid tissue damage [19,30]. The PCB, a CPC metabolite, must be the molecule responsible for pharmacological actions because it is a nucleophilic compound that firstly compensates for the disturbance in the antioxidant system [31]. However, the most important action of CPC and PCB is immunomodulatory because several anti-inflammatory mechanisms are demonstrated in several pathologies. In ethanol-induced gastric ulcers, CPC down-regulates the expression of NFκB and inflammatory cytokines such as IL-1β and TNF-α [32]. Also, CPC in carrageenan-evoked thermal hyperalgesia has been shown to be associated with lower concentrations in paw exudate of TNF-α and PGE2 by down-regulating NOS2 and COX2 [33]. Meanwhile, CPC has a positive effect against acute myocardial infarction, causing down-expression of phospho-(Ser 536)-NFκB p65 and mRNA synthesis for IL-1β, IL-6, TNF-α, and INFγ [23]. In this sense, the NFκB must participate in the pro-resolving pathways induced by CPC treatment because, in the OVA-induced asthma model, there is a similar pattern of COX2, NOS2, and MPO expression, as well as the reduction of proinflammatory cytokines. We hypothesized this because OVA-induced asthma enhances the expression of TLR4 in the lung, which is associated with damage-associated molecular patterns (DAMPS) after hard inflammation due to allergen contact of sensitized cells [34]. In inflammatory airway diseases, such as asthma and chronic obstructive pulmonary disease, inhibition of TLR4 signaling has also been suggested to be a promising therapy by reducing neutrophil recruitment and activation [35].

In this study, we show that treatment with CPC down-regulates the expression of TLR4, demonstrating that CPC could participate in inflammation resolution. Similarly, another finding that supports this idea is the prevention of endoplasmic reticulum stress by CPC treatment because there is an activation of the NLRP3 inflammasome by endoplasmic reticulum stress through the IRE1α and ATF4 pathways [36]. Similarly, another finding that supports this idea is the prevention of endoplasmic reticulum stress by CPC treatment because there is an activation of the NLRP3 inflammasome by endoplasmic reticulum stress through the IRE1α and ATF4 pathways [36,37].

The immunomodulatory character of CPC presents many facets. According to our results, CPC reduced the expression of IL-1β, INF-γ, and IL-5, as well as increased IL-4 and IL-10 through the cellular modulation of immunological cells in the lung. For example, human peripheral blood mononuclear culture cell exposure to phycocyanin up-regulated the expression of key markers for Treg cells such as Foxp3, CD25, IL-10, and TGF-β [38]. These results and the lower infiltration of inflammatory cells into perivascular and parabronchial areas observed in our histopathological study, as well as the lower expression of CD68 in pulmonary tissue due CPC treatment in OVA-induced asthma model, support the idea of a decrease in the number of macrophages in the pulmonary microenvironment, but the remaining cells may be a population of M2 macrophages, these anti-inflammatory cells are responsible for the overproduction of IL-10 and IL-4, quintessential anti-inflammatory cytokines in classical endotype T2-higer asthma. It has been demonstrated that the chromophore of CPC, the phycocyanobilin (PCB), is the responsible molecule of CPC’s physiological activity; PCB in cells is rapidly reduced to phycocyanorubin (PCR), which induces the expression of hemoxigen-ase-1 (HO-1), promoting the increase of Treg cytokine [21]. Also, HO-1 can induce the transformation of macrophages from M1 to M2, promoting pro-resolving pathways [39]. Also, if the pulmonary microenvironment in the animals treated with CPC in the model OVA-induced asthma has an anti-inflammatory state, then it is possible the production of pro-resolving molecules is enhanced because PCB does not affect the activity of lipoxygenase-5 (LOX), but CPC prevents the over-expression of COX2. In this way, LOX could promote the production of pro-resolving molecules instead of leukotrienes [40].

One of the long-term consequences of chronic pulmonary inflammation during OVA-indued asthma is airway remodeling. This dynamic process is characterized by thickening of the reticular basement membrane, increased mucus production, smooth muscle hyperplasia and hypertrophy, angiogenesis, and epithelial detachment [41,42]. The histopathological study of this research demonstrates that treatment with CPC prevents airway remodeling due to mild signs of inflammation and fibrosis, corroborated by the decreased expression of COL1A1 and trichomic Masson’s stain. The mitigation of the fibrotic and inflammatory processes by CPC treatment is associated with limiting the TLR4-NFκB-COX2 pathway and reducing bronchiolar thickening and smooth muscle hyperplasia and hypertrophy with a lesser transition from epithelial pulmonary cell to fibroblast-like cells avoiding the transformation to fibroblast and myofibroblast. Also, CPC inhibit cell proliferation, arresting cells in the G0/G1 cycle because it limits COX2 expression contributing to the reduction of the inflammatory process limiting immune cells proliferation [43].

Myofibroblasts contribute to airway remodeling in the long-term complication of asthma depending on the inflammatory microenvironment [44]; one of the principal products of these pathological cells are αSMA fibers; TGF-β1 induces transient upregulation of COL1A1 and αSMA important fibrosis markers [45]. It has been reported that CPC in vitro can reverse TGFβ1-induced EMT, up-regulating E-cadherin and down-regulating N-cadherin, vimentin, collagen type-1, and fibronectin, concluding in a low expression of TWIST, Snail, and ZEB1 transcription factors through modulating the TGFβ/SMAD signaling pathway [46,47]. The regulation of the epithelial-to-mesenchymal transition (EMT) process can contribute to pulmonary fibrosis because it changes cell-specific surface molecules, extracellular matrix components, cytoskeletal reorganization, and the activity of transcription factors [48].

Thus, CPC has a more complex action mechanism than just antioxidant and anti-inflammatory since it promotes pro-resolving inflammation pathways. In conjunction with corticosteroid action, an immunosuppressor has several secondary effects, such as long-term resistance and Cushing syndrome, that involve an alteration in metabolism and muscle atrophy [49,50].

**Figure 7:**
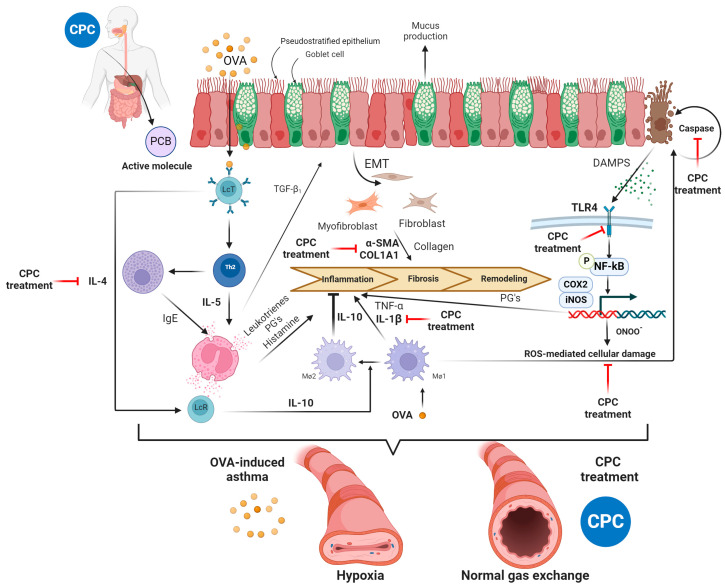
Pre-resolving pathways of C-phycocyanin in OVAinduced asthma. CPC’s antioxidant properties neutralize reactive oxygen species and free radicals avoiding tissue damage caused by caspases. OVA-induced asthma enhances the expression of TLR4 in the lung, which is associated with DAMPS, CPC also down-regulates the expression of TLR4 causing down-expression of phospho-(Ser 536)-NFκB p65 and mRNA synthesis for IL-1β and INFγ (pro-inflammation cytokines); in contrast, CPC up-regulates anti-inflammatory interleukins (IL-10 and IL-4). IL-10 over expression measures the Mø1 polarization change to Mø2 and, in conjunction with IL-4, favors a Treg response, solving inflammation. CPC also down-regulates Th2 response, avoiding IL-5 expression and consequently limiting the production of TGF-β1, preventing fibroblast and myofibroblast α-SMA and COL1A1 production, concluding in fibrosis process prevention. OVA-induced asthma promotes a state of hypoxia in the bronchioles, while the treatment with CPC favors a state of normal gas exchange. Through these mechanisms, CPC resolves inflammation, maintains lung function, and prevents the remodeling process in OVA-induced asthma. OVA: ovalbumin; CPC: C-phycocyanin; PCB: phycocyanobilin; ROS: reactive oxygen species; PG’s: prostaglandins; DAMPS: damage-associated molecular patterns; Mø1: macrophage type 1; Mø2: macrophage type 2. The red line represents the points where we propose CPC has its action, and the bold markers are the ones we measured.

A decrease in the inflammatory microenvironment in the lung, the cells associated with fibrosis, the overexpression of anti-inflammatory cytokines, and its possible role in macrophage polarization and EMT regulation led us to consider CPC’s role as an inflammation resolver more than just an antioxidant or immunomodulation molecule (Figure 7).

## 4. Materials and Methods

### 4.1. Animals

Thirty male Sprague Dawley rats (250–300 g) were acclimatized in a room controlled at 21 ± 2 °C, 40–60% relative humidity, and a 12:12 h light/dark cycle (lights on at 8 a.m.), ensuring adequate access to suitable feed and water for all animals. The experimental procedures were approved by the Institutional Bioethics Committee (ZOO-011-2022), and all procedures followed the provisions of the Official Mexican Norm [51].

Animals were randomly divided into five groups (n = 6): (1) sham + vehicle (phosphate buffer (PB) at pH 7.4 100 mM administered by oral gavage (og), (2) sham + 100 mg/Kg/d CPC og since day 10, (3) ovalbumin (OVA)-induced asthma + vehicle, (4) OVA-induced asthma + CPC, and (5) OVA-induced asthma + 5 mg/Kg/d methylprednisolone intramuscular (im) on days 7 and 14.

Bronchial asthma was induced in rats by administering twice 10 mg OVA and 300 mg aluminum hydroxide (Al(OH)_3_) in PB. The first dose was administered intraperitoneally on day one, and on the seventh day, the second dose was administered subcutaneously [52]. Al(OH)_3_ is an adjuvant to promote IgE production, and OVA is an allergen for inducing asthma crisis treatment. On days 14, 17, and 21, the OVA challenge was accomplished by administering 50 µL of 5% OVA in PBS intratracheally. This procedure was performed under sedation (35 mg/Kg sodium pentobarbital) and using 10 mg/Kg of tramadol to avoid pain. The sham group received the same procedure but used vehicles only. Two hours after the last OVA challenge, the animals were euthanized with sodium pentobarbital (90 mg/Kg ip). When the animals did not respond to painful stimuli, and before the heart was stopped (about 2–3 min), a media thoracotomy was made to obtain arterial and venous blood samples from the left and right ventricles to determine CO_2_ and lymphocytes. Then, the lungs were dissected into three portions for biochemical, molecular, and histological analyses.

### 4.2. Oxidative Stress and REDOX Environment Markers

Each lung was homogenized in 5 mL of 10 mM PB for biochemical (pulmonary MPO, caspase 3 and 9 activities) and oxidative stress markers (LP, ROS, nitrites, and GSSG) evaluation with minor modification as previously described [23,38]. Briefly, for pulmonary MPO activity, 5 μL of the homogenate was added to 160 μL of 90 mM of a citrate buffer (pH 4.5) containing 0.1% Triton X-100 and 0.65 mM of O-dianisidine. The reaction started by adding 40 μL of 0.43 mM of H_2_O_2_. The mixture was incubated at 37 °C. We monitored the absorbance at 460 nm for 5 min. The molar extinction coefficient of p-nitroaniline (ε460 = 11.3 mM^−1^cm^−1^) formed was used to express the results as mM of O-dianisidine oxidized/mg protein/min.

The lipid peroxidation assay was performed by adding 500 μL of the homogenized to 1 mL of PBS and mixing it with 7 mL of methanol: chloroform (2:1). The mixture was stirred for 30 s and kept at 4 °C for 30 min in darkness. After that time, the aqueous phase was aspirated and discarded. Then, 2 mL of the organic phase was utilized for fluorescence measurement at 370 nm of excitation and 430 nm of emission using an RF5000U Shimadzu Spectrophotometer. The results are expressed as relative fluorescence units (RFU) per milligram of protein.

For the quantification of ROS, 10 μL of homogenized was combined with 1940 μL of TRIS: HEPES (18:1, *v*/*v*) and 50 μL of 2′,7′-dichlorofluorescine diacetate (DCFH-DA, 0.2 mg/mL in methanol). The mixture was then incubated in a water bath at 37 °C for one hour. Then, the reaction was stopped by freezing, and the fluorescence was measured at 488 nm of excitation and 525 nm of emission using an RF5000U Shimadzu Spectrophotometer. The results are expressed as ng of 2′,7′-dichlorofluorescein (2′,7′-DCF) formed per mg of protein per hour.

Nitrites (NO_2_) were determined by a Griess reaction. Briefly, 100 μL of the homogenate was mixed with 250 μL of 36% HCl and 250 μL of a 20% metallic zinc suspension for reducing nitrates. The mixture was incubated at 37 °C for one hour, followed by centrifugation at 4000× *g* for 2 min. Then, 50 μL of the supernatant was transferred to a 96-well plate along with 100 μL of Griess reagent. After incubating for 15 min at room temperature, the absorbance at 530 nm was measured. Results are presented as μg of NO_2_ per mg of protein.

For GSH content, 500 μL of the homogenized was mixed with 100 μL of 30% phosphoric acid. After centrifugation at 19,000× *g* for 30 min at 4 °C, 30 μL of the supernatant was added to 1870 μL of FEDTA (PBS with 5 mM EDTA at pH 8.0) for GSH quantification. Finally, 100 μL of o-phthaldialdehyde (1 mg/mL solubilized in methanol grade HPLC) was added to evaluate fluorescence (350 nm for excitation and 420 nm for emission) after 10 min of incubation. The results are expressed as mg GSHG per milligram of protein.

Caspase 3 and 9 activities were assessed using a colorimetric assay detected by cleavage of p-nitroaniline (pNA) from the DEVD-pNA (caspase 3) or LEHD-pNA (caspase 9) at 405 nm. Briefly, 10 μL of homogenate was added to a 96-well polystyrene plate mixed with 85 μL of caspase buffer containing 20 mM HEPES, 0.1% CHAPS, 5 mM DTT and 2 mM EDTA, pH 7.4. The reaction was initiated when 10 μL of substrate DEVD-pNA or LEHD-pNA. The mix was incubated at 37 °C, and absorbance at 405 nm was monitored for 1 h. Caspase activity is expressed as pmols of PNA released/mg protein/h.

All techniques that used protein quantification were performed using 1 μL of homogenate mixed with 99 μL deionized water and 900 µL of Bradford reagent. The mixture was incubated at room temperature for 10 min, and absorbance was measured at 595 nm.

### 4.3. Biochemical, Histological, and Molecular Assays

For the functional pulmonary test associated with bronchial asthma, CO_2_ was determined using a RANDOX kit. Meanwhile, lymphocyte count was made manually using the Nue Bauer chamber employing Turk liquid in a double-blind assay.

For myeloperoxidase (MPO) activity, 2 μL of the homogenate was added to 160 μL of 90 mM of a citrate buffer (pH 4.5) containing 0.1% Triton X-100 and 0.65 mM of O-dianisidine. The reaction started with the addition of 40 μL of 0.43 mM of H_2_O_2_. The mixture was incubated at 37 °C. We monitored the absorbance at 460 nm for 15 min, and at 460 nm for 5 min. The molar extinction coefficient of p-nitroaniline formed was used to express the results as mmoles O-dianisidine oxidized/mg protein/min (ε460 = 11.3 mM^−1^cm^−1^).

For western blotting, 100 μL of the homogenate (n = 3 per group) was combined with 100 μL of a complete protease inhibitor cocktail (Santa Cruz Biotechnology, Dallas, TX, USA) and subjected to a 3 min incubation in a boiling water bath. Electrophoresis was carried out by loading 50 µg protein samples per lane onto a 10 or 15% SDS-PAGE gel and separating them at a constant voltage of 100 V for one hour. The proteins were then electro transferred to PVDF membranes (Millipore, Bedford, MA, USA) using a Trans-Blot Turbo System (Bio-Rad, Hercules, CA, USA) at 25 V and 2.5 A for 10 min. Subsequently, membranes were blocked with PBST (PBS with 0.05% Tween 20 and 5% low-fat MilkSvelty^®^) for 1 h under constant agitation at room temperature. The membranes were incubated overnight at 4 °C in PBST with primary antibodies (Santa Cruz Biotechnology, Dallas, TX, USA) diluted 1:1000 for αSMA (sc-390453), COL1A1 (sc-293182), TLR4 (sc-293072), CD68 (sc-20060), p-NFkB p65 (sc-101752), COX2 (sc-23983) and diluted 1:500 for NOS2 (sc-7271).

Following incubation, the membranes were washed three times with fresh PBST (30 min per wash) and then incubated for one hour under constant agitation at room temperature with a specific secondary antibody linked to HPR (Santa Cruz Biotechnology, Dallas, TX, USA) diluted 1:3000. Subsequently, the membranes underwent three additional washes with fresh PBST (30 min per wash). Finally, protein bands were chemiluminescence revealed on photographic plates, using Luminata™ Forte^®^ (Millipore, Billerica, MA, USA). β-actin (Santa Cruz Biotechnology, Dallas, TX, USA; sc-47778, diluted: 1:1500) was used as constitutive protein expression and loading control. The optical density (O.D.) from protein bands was analyzed by ImageJ/FIJI (1.46v. NIH, Bethesda, Rockville, MD, USA), and the results are presented as the O.D. of protein/O.D. of β-actin ratio.

For the histological analysis, one portion of the lung was fixed in 4% paraformaldehyde in PBS for 48 h and embedded in paraffin. We obtained slices of 7 μm with a standard microtome stained with hematoxylin-eosin, Masson’s trichromic, and periodic acid–Schiff-blue toluidine techniques.

For qPCR cytokine determination, each sample was frozen in 500 μL of TRIzol (Invitrogen, Waltham, MA, USA) at −80 °C until RNA extraction. According to the supplier’s specifications, macerated samples were recovered in 1 mL sterile microtube for total RNA extraction by the TRIzol method. RNA integrity was verified on a 1.5% agarose gel. For retrotranscription (RT), 3 μg of total RNA from each sample was used, to which 0.5 μg of oligo (dT) (Invitrogen) was added and incubated at 70 °C for 10 min. To obtain the cDNA, a master mix containing 1X single strand buffer, 0.5 mM DTT, 500 mM of each deoxynucleotide triphosphate (dNTP), and 200 U of MMLV reverse transcriptase (Thermo Scientific, Waltham, MA, USA). The RT reactions were incubated at 42 °C for one hour. The cDNA was stored at −20 °C until use. Real-time PCR was performed to measure the relative expression of cytokines using 100 ng of cDNA, a Master Mix with 1.5 mM MgCl_2_ (Ampliqon III), and Eva Green (Biotium, Fremont, CA, USA). In addition, ROX (Sigma-Aldrich, St. Louis, MO, USA) was used as a reference fluorochrome. All reactions were performed using the Step One Plus qPCR kit (Applied Biosystems, Waltham, MA, USA). The actin gene was used as an endogenous control.

The specific primers for each cytokine tested are indicated in Table 1.

### 4.4. Statistical Analysis

For all variables, the results are expressed as the mean + standard error, and they were evaluated using a two-way analysis of variance and Student–Newmann–Keuls post hoc test. The factors analyzed were asthma presence and treatment. *p* < 0.05 were considered statistically significant.

## 5. Conclusions

C-phycocyanin prevents oxidative stress and inflammation and improves lung tissue remodeling inherent to the pathophysiology of asthma. These beneficial effects rely on CPC preventing OVA-induced pulmonary oxidative stress, promoting the pro-resolving pathways through regulating Th2 responses. Therefore, CPC has the potential to be used for the co-treatment of asthma as an alternative therapy. However, further research is needed to better understand CPC’s molecular mechanism of action before its development as a pro-pharmaceutical product for human consumption.

## Figures and Tables

**Table 1:** Primer’s specifications.

Gene	Primers	Product Length (BP)	Annealing Temperature
*IL-1β*	5′-agcccatcctctgtgactcatg-3′ 5′-gctgatgtaccagttggggaac-3′	104	60.5 °C
*INF-γ*	5′-ttttgcagctctgcctcat-3′ 5′-agcatccatgctacttgagttaaa-3′	106	63 °C
*IL-10*	5′-tgccaagccttgtcagaaathg3′ 5′-tgagtgtcacgtaggcttcta-3′	386	58 °C
*IL-5*	5′-aactctcagctgtgtctgggc-3′ 5′-gacttccattgcccactctgta-3′	367	59 °C
*IL-4*	5′-accttgctgtcaccctgttc-3′ 5′-gttgtgagcgtggactcattc-3′	352	60.5 °C
*β-Actin*	5′-agcccatcctctgtgactcatg-3′ 5′-gctgatgtaccagttggggaac-3′	422	63 °C

## Data Availability

The datasets generated during the current study are available from the corresponding author upon reasonable request.
